# Evidence of improving antiretroviral therapy treatment delays: an analysis of eight years of programmatic outcomes in Blantyre, Malawi

**DOI:** 10.1186/1471-2458-13-490

**Published:** 2013-05-21

**Authors:** Derek J Sloan, Joep J van Oosterhout, Ken Malisita, Eddie M Phiri, David G Lalloo, Bernadette O’Hare, Peter MacPherson

**Affiliations:** 1 Malawi-Liverpool-Wellcome Trust and Liverpool School of Tropical Medicine, Chichiri 3, PO 30096, Blantyre, Malawi; 2 Centre for Global Health and Infection, University of Liverpool, Liverpool, UK; 3 College of Medicine, University of Malawi, Mahatma Ghandi Road, Blantyre, Malawi; 4 Faculty of Infectious and Tropical Diseases, Department of Clinical Sciences, Keppel Street, London, UK; 5 Clinical Group, Liverpool School of Tropical Medicine, Pembroke Place, Liverpool L3 5QA, UK

**Keywords:** HIV, Antiretroviral therapy, Linkage to care, HIV testing and counselling, Africa, Programmatic research

## Abstract

**Background:**

Impressive achievements have been made towards achieving universal coverage of antiretroviral therapy (ART) in sub-Saharan Africa. However, the effects of rapid ART scale-up on delays between HIV diagnosis and treatment initiation have not been well described.

**Methods:**

A retrospective cohort study covering eight years of ART initiators (2004–2011) was conducted at Queen Elizabeth Central Hospital (QECH) in Blantyre, Malawi. The time between most recent positive HIV test and ART initiation was calculated and temporal trends in delay to initiation were described. Factors associated with time to initiation were investigated using multivariate regression analysis.

**Results:**

From 2004–2011, there were 15,949 ART initiations at QECH (56% female; 8% children [0–10 years] and 5% adolescents [10–20 years]). Male initiators were likely to have more advanced HIV infection at initiation than female initiators (70% vs. 64% in WHO stage 3 or 4). Over the eight years studied, there were declines in treatment delay, with 2011 having the shortest delay at 36.5 days. On multivariate analysis CD4 count <50 cells/μl (adjusted geometric mean ratio [aGMR]: aGMR: 0.53, bias-corrected accelerated [BCA] 95% CI: 0.42-0.68) was associated with shorter ART treatment delay. Women (aGMR: 1.12, BCA 95% CI: 1.03-1.22) and patients diagnosed with HIV at another facility outside QECH (aGMR: 1.61, BCA 95% CI: 1.47-1.77) had significantly longer treatment delay.

**Conclusions:**

Continued improvements in treatment delays provide evidence that universal access to ART can be achieved using the public health approach adopted by Malawi However, the longer delays for women and patients diagnosed at outlying sites emphasises the need for targeted interventions to support equitable access for these groups.

## Background

Since 2003, there have been tremendous global achievements in scaling up delivery of antiretroviral therapy (ART) for people infected with HIV, with over 6.6 million people now receiving treatment [1]. The most impressive gains have been seen in sub-Saharan Africa. In 2009, 3.9 million people in the region were taking ART but by the end of 2011, more than 5.1 million people had been initiated onto ART, a 27% increase [2].

Malawi, one of the poorest countries in the world, has pioneered the public health approach to ART delivery [3,4]. The public health approach involves decentralisation of ART delivery to primary health care clinics, task-shifting from clinicians to nurses and counsellors, and a reporting system based on collection of facility-level aggregate statistics that allow clear analysis of trends in HIV testing data, uptake of ART and outcomes of ART initiators [5]. By mid-2011, more than 400,000 HIV-infected Malawians had initiated ART through the National Programme [6].

Although the rapid scaling up of ART programmes have been impressive in their speed, impact upon population mortality and on reducing HIV incidence [2], there are increasing concerns about the effect that an ever-expanding number of patients requiring life-long clinical management will have on staff workload and ART clinic sustainability. Moreover, a recent systematic review has highlighted the high rate of attrition in the pre-ART period [7], with long delays and failures to complete eligibility assessments (CD4 counts and WHO clinical staging assessments) major contributors to sub-optimal retention before initiation [8]. Alternatively, as ART guidelines are expanded to include individuals with higher CD4 counts, treatment delays may be shortened.

This study examined changing patterns in length of time between diagnosis of HIV infection and initiation of antiretroviral therapy (“ART treatment delay”) at the largest single public health facility providing ART in Malawi over eight years. The objectives were to describe temporal trends in ART treatment delay and investigate factors associated with increased risk of delay since ART became available free-of-charge at the clinic.

## Methods

### Study design

A retrospective cohort study.

### Study site and population

Queen Elizabeth Central Hospital (QECH) is the largest public health facility in Malawi and is a referral centre for patients in the Southern Region of the country. The ART clinic at QECH was established as a fee-paying clinic in 2000 and since 2004, ART has been provided free-of-charge according to the National Ministry of Health scale-up programme. The QECH ART clinic profile and outcomes of patients initiating ART have been described in detail previously [9].

Clinical and socio-demographic details of all ART initiators have been prospectively entered into the clinic’s electronic data management system [10] since 2008. Records of ART initiators before this date have been retrospectively captured and validated.

**Figure 1:**
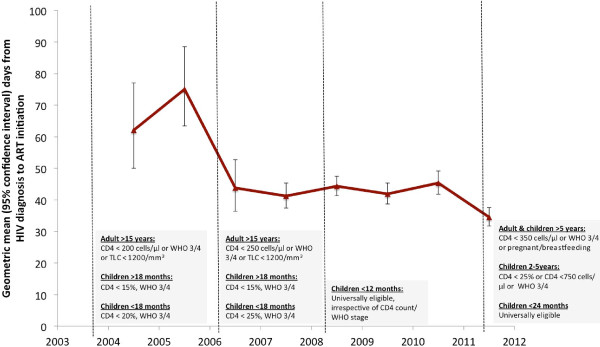
**Temporal trends in delay from HIV diagnosis to ART initiation at Queen Elizabeth Central Hospital, Blantyre, Malawi: 2004–2011, and evolution of national treatment guidelines.** TLC: total lymphocyte count.

From 2003 until 2006, Malawi National ART eligibility criteria for adults (≥15 years) were presence of a WHO stage 3 or 4 defining illness, or a CD4 count of <200 cells/μl, or a total lymphocyte count (TLC) in an individual in WHO stage 2 of <1200 cells/μl (Figure 1). The CD4 count threshold was revised upwards to a cut-off of <250 cells/μl in 2006 and to <350 cells/μl in September 2011.

For children less than 15 years old ART initiation guidelines in Malawi have evolved in line with the publication of new evidence over the years. From, 2003 to 2006, children younger than 18 months old who were eligible for ART if their CD4 percentage was < 25% or they were in WHO paediatric stage 3 or 4. Children aged older than 18 months if their CD4 percentage was < 15%. As new evidence became available the guidelines were updated and the thresholds for starting ART in asymptomatic children lowered. In 2008 national guidelines were updated to recommend that all children aged younger than 12 months with a confirmed or presumed diagnosis of HIV were universally eligible for ART, regardless of immune status. This was extended to all children younger than 24 months in September 2011.

In this study, we extracted data from the ART clinic database for the period from January 2004 to December 2011 when ART was available free-of-charge. Individuals who initiated ART between 2004 and 2011 (regardless of date of HIV diagnosis) were eligible for inclusion.

### Defining ART treatment delay

Cohort entry was defined as the date of most recent positive HIV test recorded in the ART clinic database. Cohort exit was defined as the date of ART initiation. Where either the date of last positive HIV test or the date of ART initiation was missing from the database, the patient was not included in the analysis.

### Statistical methods

Statistical analysis was conducted using Stata/IC 11.2 (College Station, Texas, USA). Baseline characteristics of ART initiators were stratified by sex and compared using Pearson’s chi-square tests for categorical variables and Student’s *t* test for continuous variables. Children were defined as being between 0 and 10 years at ART initiation and adolescents between 10 and 20 years at initiation.

Number of days between last positive HIV test and initiation of ART showed a highly skewed distribution and results were therefore transformed to log_e_ before analysis. Mean number of days to ART initiation was compared between groups and by cohort year using linear regression with back-transformation of regression coefficients to obtain geometric mean ratios (GMRs). As some results remained skewed after log_e_ transformation, analyses were bootstrapped with 10,000 repetitions to estimate bias-corrected accelerated confidence intervals. Variables considered as *a priori* confounders (age, sex, CD4 count, year of initiation), and variables that were significant at the p < 0.10 level on univariate analysis, were included in the adjusted multivariate model.

### Ethical considerations

The College of Medicine of Malawi Research Ethics Committee granted ethical approval for this study. As this was a retrospective study analysing routinely collected data, individual consent was not sought. All patient identifiers were removed prior to analysis.

## Results

### Baseline characteristics

Between January 2004 and December 2011, there were 15,949 ART initiations at the QECH ART Clinic, with more females (8,988, 56.4%) than males (6,961, 43.6%) starting treatment. Characteristics of the ART initiators, stratified by sex, are shown in Table 1. Male initiators were older than female initiators (median: 34 years, interquartile range [IQR]: 27–42 vs. 31 years, IQR: 25–38; p < 0.001) and were more likely to have to have advanced HIV infection at initiation: 4,876/6,961 (69.9%) of male initiators were in stage 3 or 4 compared to 5,724/8,988 (63.7%) of female initiators (p < 0.001); and a greater proportion of male initiators (533/3,321, 16.1%) had a CD4 count of <50 cells/μl at initiation compared to female initiators (528/4,427, 11.9%).

**Table 1:** Baseline characteristics of ART initiators at Queen Elizabeth Central Hospital between 2004 and 2011

**Variable**	**Female**	**%**	**Male**	**%**	**P-value**
Total	8,988		6,961		
Median age at initiation (years); IQR	31	25-38	34	27-42	<0.001
Age group at initiation					
0-5 years	475	5.3	537	7.8	<0.001
5-15 years	583	6.5	521	7.5	
15-25 years	1,042	11.7	335	4.9	
25-35 years	3,715	41.6	2,033	29.4	
35-45 years	2,077	23.3	2,091	30.3	
≥ 45 years	1,040	11.6	1,387	20.1	
*Site of HIV test*					
QECH	3,023	33.6	2,361	33.9	0.860
Outside QECH	4,140	46.1	3,176	45.6	
Unknown	1,825	20.3	1,424	20.5	
*WHO stage at initiation*					
Stage 1 or 2	3,264	36.3	2,094	30.1	<0.001
Stage 3 or 4	5,724	63.7	4,867	69.9	
*CD4 count* (*cells*/*μl*)^*^					
≥ 350	167	3.8	103	3.1	<0.001
250 – 350	1,013	22.9	755	22.7	
50 – 250	2,719	61.4	1,930	58.1	
<50	528	11.9	533	16.1	
*Year of ART initiation*					
2004-2005	562	6.3	404	5.8	<0.001
2005-2006	1,208	13.4	801	11.5	
2006-2007	1,250	13.9	887	12.7	
2007-2008	1,253	13.9	1,010	14.5	
2008-2009	1,249	13.9	964	13.9	
2009-2010	1,134	12.6	1,024	14.7	
2010-2011	1,154	12.8	1,002	14.4	
2011-2012	1,178	13.1	866	12.4	
Median time (days) between last positive HIV test and initiation of ART; IQR	36	14-120	33	12-94	0.001

*CD4 count measured in 4,427 women and 3,321 men. Denominator for this variable is number of men and women who had CD4 count measured.

There were no differences in the site of the last positive HIV test, with a similar proportion of males (3,176/6961, 45.6%) and females (4,140/8,988, 46.1%) testing at sites outside of QECH (p = 0.860).

### Child and adolescent ART initiators

Similar proportions of child (0–10 years) ART initiators were male (884/1,694, 52.2%) and female (810/1,694, 47.8%). However, adolescent (10–20 years) initiators were significantly less likely to be male than female (342/856 [40.0%] vs. 514/856 [60.0%]). Adolescents had the highest proportion of ART initiators who were in WHO stage 3 or 4 (614/586, 71.7%) compared to children (1,089/1,694, 64.3%) and adults older than 20 years (8,888/13,399, 66.3%). Additionally, adolescents (58/380, 15.3%) were most likely to initiate ART with a CD4 count of <50 cells/μl compared to children (33/666, 5.0%) and adults (970/6,702, 14.5%).

### Temporal trends in delay from last positive HIV test to initiation of ART

In total, 9,511/15,949 (59.6%) ART initiators had complete data for date of ART initiation and date of most recent positive HIV test with the remaining 40.4% of individuals having missing data for either date of ART initiation, HTC or both. There were no differences between individuals with missing and non-missing outcome data in terms of age group, sex and CD4 count strata. However, individuals initiating ART at QECH were less likely to have missing date of HIV diagnosis than individuals initiating ART at another clinical site. The overall median treatment delay in the group with complete data for delay in ART initiation was 35 days (IQR: 13–107).

Men (33 days, IQR: 12–94) had a shorter median ART treatment delay compared to women (36 days, IQR: 14–120; p = 0.001). There were slightly longer median time to initiation for children (40 days, IQR: 14–114) and adolescents (39 days, IQR: 13–127) compared to adults (34 days, IQR: 13–105); p < 0.001.

In the years immediately following introduction of free ART in the clinic, there was a trend towards increasing ART treatment delay (Figure 1), with a peak in 2005 (geometric mean: 67.3 days, 95% CI: 58.9 – 76.8). However between 2005 and 2007, there was a rapid decline in ART treatment delay to a lowest geometric mean of 43.3 days (95% CI: 39.7 – 47.3). Between 2007 and 2010, there was a slow upwards trend in treatment delay, but followed by a sharp decline in 2011, where the geometric mean number of days of ART treatment delay was 36.5 days (95% CI: 33.7-39.6).

### Clinical and temporal associations with time from last positive HIV test to initiation of ART

On univariate analysis (Table 2), female initiators experienced longer treatment delays (geometric mean ratio [GMR]: 1.13, bias corrected accelerated [BCA] 95% CI: 1.06-1.21) compared to male initiators. Younger children in the 0–5 years age group had shorter treatment delays (GMR: 0.87, BCA 95% CI: 0.75-0.99), compared to adults in the 25–35 years age group, whereas older children and adolescents in the 5–15 year age group were had a longer delays (GMR: 1.16, BCA 95% CI: 0.99-1.37).

**Table 2:** Univariate and multivariate associations with time from last positive HIV test and initiation of ART

**Variable**	**GMR**^**±**^	95% CI^*^	**Adjusted GMR**^**±**^	95% CI^*^	**P**
*Sex*					
Men	1		1		0.007
Women	1.13	1.06-1.21	1.12	1.03-1.22	
*Age group*					
0-5 years	0.87	0.75-0.99	1.02	0.84-1.24	0.526
5-15 years	1.16	0.99-1.37	1.14	0.93-1.39	
15-25 years	1.00	0.89-1.23	0.93	0.81-1.08	
25-35 years	1		1		
35-45 years	1.00	0.92-1.08	0.96	0.87-1.06	
≥ 45 years	1.00	0.90-1.10	0.96	0.84-1.09	
*Site of HIV test*					
QECH	1		1		
Facility other than QECH	1.65	1.54-1.77	1.61	1.47-1.77	<0.001
Unknown	1.82	1.64-2.02	1.93	1.66-2.24	
*WHO stage at registration*					
Stage 1 or 2	1				
Stage 3 or 4	1.01	0.95-1.08			
*CD4 count* (*cells*/*μl*)					
≥ 350	1		1		<0.001
250 – 350	0.87	0.70-1.11	0.96	0.75-1.20	
50 – 250	0.76	0.61-0.95	0.77	0.62-0.98	
<50	0.50	0.40-0.63	0.53	0.42-0.68	
*Year of ART initiation*					
2004	0.92	0.75-1.14	0.79	0.52-1.21	0.004
2005	1		1		
2006	0.72	0.60-0.87	0.75	0.48-1.16	
2007	0.64	0.55-0.76	0.71	0.50-1.03	
2008	0.68	0.59-0.80	0.72	0.51-1.02	
2009	0.69	0.59-0.80	0.69	0.49-0.99	
2010	0.75	0.64-0.88	0.85	0.60-1.21	
2011	0.54	0.47-0.63	0.84	0.59-1.21	
*Number of ART initiators per year* (*continuous*)	1.00	1.00-1.00			

^±^Bootstrapped estimates of geometric mean ratios, adjusted for sex, age group, site of HIV test, CD4 count strata and year of ART initiation.

^*^Bias corrected accelerated 95% confidence intervals.

*GMR* Geometric mean ratio, *CI* confidence interval, *QECH* Queen Elizabeth Central Hospital.

ART initiators whose last positive test for HIV was at a clinical facility outside of QECH (any other HIV care facility) were more likely to experience delay (GMR: 1.65, BCA 95% CI: 1.54-1.77). Whereas advanced WHO clinical stage (stage 3 or 4) was not associated with delay (GMR: 1.01, BCA 95% CI: 0.95-1.08), individuals with an initiation CD4 count of <50 cells/μl (GMR: 0.50, BCA 95% CI: 0.40-0.63) and 50–200 cells/μl (GMR: 0.76, BCA: 0.61-0.95) had significantly lower treatment delay than individuals with a CD4 count of ≥ 350 cells/μl.

When examined by year, a trend of significantly declining ART treatment delay compared to the year with the longest time from HIV-positive test to ART initiation (2005) was seen (test for trend p < 0.001). The lowest risk of delay was in 2011 (GMR: 0.54, BCA 95% CI: 0.47-0.63).

On multivariate adjusted analysis, women remained more likely to experience a longer ART treatment delay than men (adjusted GMR [aGMR]: 1.12, BCA 95% CI: 1.03-1.22), however there was no significant association between age group at initiation and delay (p = 0.526). Individuals whose last positive HIV test was outside of QECH had a longer delay to ART initiation (aGMR: 1.61, BCA 95% CI: 1.47-1.77). Lower CD4 count remained significantly associated with shorter delay to initiation, with individuals in the <50 cells/μl group (aGMR: 0.53, BCA 95% CI: 0.42-0.68) and the 50–200 cells/μl group (aGMR: 0.77, BCA 95% CI: 0.62-0.98) compared to those in the ≥350 group. There remained a non-significant trend towards shorter delay over the years studies (test for trend: p = 0.089).

## Discussion

The main finding from the study was the evidence of sustained improvements in ART treatment delay from most recent positive HIV test to initiation of ART over eight years of rapid programmatic scale-up, a period during which nearly 16,000 individuals initiated treatment. The most recent year that was analysed (2011) had the shortest mean treatment delay, suggesting that the public health approach to ART delivery [3] that is recommended in the Malawian National Programme and implemented at QECH, continues to meet its objective of providing rapid access to ART despite extremely limited resources.

In September 2011, new Malawian National ART guidelines were introduced promoting initiation of ART for HIV-infected individuals with a CD4 count of <350 cells/μl, or in WHO stage 3 or 4, or who are pregnant or breastfeeding [11]. Given the long experience of providing ART with no appreciable increase in delay seen in this study, we anticipate that time to initiation will continue to shorten – although with a greater number of potential patients meeting eligibility criteria, current levels of resourcing will need to be sustained or increased. This study found an overall median treatment delay of 35 days, although this varied cover time and was considerably shorter in more recent years. This compares similarly with other national programmes in the region: a recent systematic review found that the majority of ART initiators started treatment within one month [12], although two studies reported higher median delays of 2.4 months [13] and 6.6 months [14].

A key success to the sustainability of the Malawian national programme has been decentralisation of ART services from tertiary facilities to primary health care centres [15]. However, we found that patients who were diagnosed with HIV at a site outside of QECH and subsequently attended QECH for ART initiation were significantly more likely to experience delay. This may have been because, unlike patients who were diagnosed within QECH, they would have had to make repeated journeys to the hospital for ART eligibility assessments and treatment education classes [16]. Further, patients who were diagnosed at a primary care facility and opted to initiate ART at QECH may have had more complex clinical problems requiring specialist care that delayed ART initiation. Alternatively, they may have been reluctant to return to the primary health care centre for treatment because of anticipated stigma or previous bad experiences with providers [17]. Finally, individuals diagnosed with HIV at the tertiary hospital may have had more advanced immunosuppression, which mean that CD4 eligibility thresholds would be met earlier.

Women had longer delay to ART initiation than men, possibly reflecting their propensity to be diagnosed earlier in the course of their infection through routine HTC during antenatal care [8,18]. Men are known to initiate ART at a more advanced stage of illness and with lower CD4 counts than women [19] and have a higher risk of immediate post-ART initiation mortality [20,21]. In this study, we were not able to adjust for the effect of pregnancy (which is associated with diagnosis at an earlier stage of HIV infection [8]) at ART initiation to determine whether this could have explained women’s longer delay. Nevertheless, given that more women currently access ART in sub-Saharan Africa [2], further studies, including qualitative research, are required to understand the gendered factors contributing to delay between HIV diagnosis and initiation of ART.

Factors resulting in delay between HIV diagnosis and initiation of ART can include the complexity and time-consuming nature of eligibility assessments (CD4 count measurement and WHO clinical staging) [8,14,22], especially when these require substantial patient expenditure and repeat facility visits [23]. Moves towards introducing point-of-care CD4 count measurement [24] within HIV testing sites should allow same-day, same-clinic eligibility assessments reducing delays for men and women, while the universal ART eligibility of pregnant women infected with HIV in Malawi (“Option B+”) [25] should result in more rapid initiation of treatment for pregnant women. We noted peaks in ART initiations in 2004 and 2005, after ART became freely available at the clinic through the national treatment programmes. There are two possible related reasons for this. Firstly, the large number of individuals who were awaiting treatment could have overwhelmed clinic capacity. However, we found that the number of clients initiating ART per year was not significantly associated with delay. Alternatively with treatment guidelines stipulating that only individuals with advanced immunosuppression (CD4 count <200 cells/ μl were eligible for ART during this time, overall HIV diagnosed individuals may have had to wait longer before they met ART eligibility criteria.

The majority of ART initiators in this study were adolescents and young adults. Adolescents were significantly more likely to initiate ART with advanced HIV infection than individuals in other age groups. This supports data from Zimbabwe showing that HIV-infected adolescents are a neglected group, with high rates of undiagnosed HIV [26], late presentation for care with high a prevalence of chronic complications [27,28] and numerous socio-economic complications, including an extremely high rate of orphanhood of 73% [28] and emotional and psychological difficulties. We were reassured to find that, on adjusted analysis, adolescents and young adults did not have a higher risk of ART treatment delay, and this may be due to the availability of teen groups and integrated family clinics at QECH supporting access to care. However, the advanced stage of HIV at which the majority of children and adolescents are diagnosed requires urgent intervention, perhaps by including HIV screening as part of regular infant health screening clinics.

There were limitations to this study. We only examined delay from most recent positive HIV test to initiation of ART and some individuals may have tested positive on a prior occasion. Nearly 40% of ART initiators did not have complete data recorded for either date of most recent HIV-positive test or ART initiation, meaning that treatment delay may have been under- or over-estimated. As this was a retrospective cohort study (although using prospectively collected data), we were unable to assess several unrecorded variables which may have potentially impacted upon delay. Finally, we did not give a time-delineated definition of “delay” and instead used a continuous dependent variable within our regression models. This avoids over-simplification of a complex construct and recognises that criteria for ART initiation have evolved over time. However, it means that we cannot give a definite proportion of patients who were “delayed”. New Malawian National guidelines [11] introduced in September 2011 recommend that ART is initiated within seven days of confirmed infection (for those meeting eligibility criteria). Even in the year with the shortest mean delay to treatment initiation (2011), only 18% of ART initiators initiated treatment within 7 days of their most recent positive HIV test. It will be important to continue to measure ART delays with regards to this target.

## Conclusion

In conclusion, this study reports on substantial improvements in ART treatment delay in the largest facility providing care in Malawi, despite rapid programmatic scale-up over eight years. Although these achievements are impressive in the context of an extremely resource-limited setting, a considerable decrease in treatment delay is still required, especially where “test-and-treat” approaches to ART are being considered. The increased risk of ART treatment delay among women emphasises the need to ensure equity in access to care.

## Competing interests

The authors declare that they have no competing interests.

## Authors’ contributions

DJS, PM, JvO: Designed the study. EMP: Collected the data. PM: Undertook statistical analysis. DJS, PM, BoH, JVO, DGL: Wrote the first draft of the manuscript. All authors reviewed and approved the final manuscript.

## Pre-publication history

The pre-publication history for this paper can be accessed here:

http://www.biomedcentral.com/1471-2458/13/490/prepub
